# Preparation and Characterization of a Novel Sulfonated Titanium Oxide Incorporated Chitosan Nanocomposite Membranes for Fuel Cell Application

**DOI:** 10.3390/membranes11060450

**Published:** 2021-06-17

**Authors:** Saad Ahmed, Tasleem Arshad, Amir Zada, Annum Afzal, Muhammad Khan, Amjad Hussain, Muhammad Hassan, Muhammad Ali, Shiai Xu

**Affiliations:** 1 School of Materials, East China University of Science and Technology, Shanghai 200237, China; saad_ahmad02@yahoo.com (S.A.); ali1095@gmail.com (M.A.); 2 School of Chemical Engineering, Qinghai University, Xining 810016, China; 3 Department of Chemistry, University of Okara, Okara 56300, Pakistan; tasleemarshad92@gmail.com (T.A.); anumafzal@mail.ustc.edu.cn (A.A.); mkhanchemistry@yahoo.com (M.K.); amjadhussain@uo.edu.pk (A.H.); mhassan@szu.edu.cn (M.H.); 4 Department of Chemistry, Abdul Wali Khan University Mardan, Mardan 23200, Pakistan; amistry009@yahoo.com

**Keywords:** sulfonated TiO_2_, chitosan, nanocomposite membrane, proton exchange membrane fuel cell

## Abstract

In this study, nano-TiO_2_ sulfonated with 1,3-propane sultone (STiO_2_) was incorporated into the chitosan (CS) matrix for the preparation of CS/STiO_2_ nanocomposite membranes for fuel cell applications. The grafting of sulfonic acid (–SO_3_H) groups was confirmed by Fourier transform infrared spectroscopy, thermogravimetric analysis and energy-dispersive X-ray spectroscopy. The physicochemical properties of these prepared membranes, such as water uptake, swelling ratio, thermal and mechanical stability, ion exchange capacity and proton conductivity, were determined. The proton conducting groups on the surface of nano-TiO_2_ can form continuous proton conducting pathways along the CS/STiO_2_ interface and thus improve the proton conductivity of CS/STiO_2_ nanocomposite membranes. The CS/STiO_2_ nanocomposite membrane with 5 wt% of sulfonated TiO_2_ showed a proton conductivity (0.035 S·cm^−1^) equal to that of commercial Nafion 117 membrane (0.033 S·cm^−1^). The thermal and mechanical stability of the nanocomposite membranes were improved because the interfacial interaction between the -SO_3_H group of TiO_2_ and the –NH_2_ group of CS can restrict the mobility of CS chains to enhance the thermal and mechanical stability of the nanocomposite membranes. These CS/STiO_2_ nanocomposite membranes have promising applications in proton exchange membrane fuel cells.

## 1. Introduction

Increasing environmental pollution and depleting natural resources have emphasized the need for clean and sustainable energy [1,2,3,4,5]. One of the most effective methods for this purpose is the conversion of chemical energy into electrical energy [6,7,8,9]. In recent years, proton exchange membrane fuel cells (PEMFCs) have gained much consideration in energy fields owing to their high effectiveness and environmental friendliness [10,11,12,13]. Perfluorosulfonic polymer-based membranes such as Nafion have high proton conductivity and notable mechanical and chemical stability due to the particular structure of Nafion [14,15,16,17]. However, the practical applicability of Nafion is restricted by its high price and sudden decline in proton conductivity at temperatures higher than 100 °C. Chitosan (CS) has the advantages of low cost, hydrophilicity and environmental benefits [18,19,20], making it attractive for various applications such as food packaging, drug delivery and PEMs [21,22]. However, its proton conductivity is much lower than that of Nafion, which represents a major hindrance to the applications of CS-based membranes.

Organic/inorganic composites are composed of the polymer matrix and inorganic fillers [23], and they have been widely utilized in different fields such as ultrafiltration, pervaporation, dye-sensitized solar cells, lithium-ion batteries and biosensors [24,25,26,27,28]. Various inorganic fillers, such as TiO_2_, zirconium oxide, halloysite, and graphene oxide, can be introduced into the polymer matrix for the fabrication of PEMs [29,30,31,32], which can improve the mechanical and thermal stabilities of PEMs, as well as the proton conductivity in some cases [33]. Incorporating acid-grafted inorganic fillers into the polymer matrix can increase the proton conductivity and interfacial compatibility of the composite membranes [34,35,36,37,38]. TiO_2_ as inorganic fillers has been widely used in photo-catalysis, biosensors and solar cells due to its hydrophilic nature and ultraviolet resistance [39,40,41,42,43]. Earlier reported literature narrates that incorporating TiO_2_ as an inorganic filler has a positive effect on the thermal and mechanical stabilities of the resultant membranes [44,45]. Slade et al. reported that the addition of TiO_2_ in Nafion 1100 membranes improved the mechanical stability and ion exchange capacity of the Nafion 1100 membranes [46]. Similarly, the hygroscopic nature of TiO_2_ aids in water management within membranes and subsequently improves proton conductivity [47].

In this study, a novel sulfonated TiO_2_ was prepared and embedded into the CS matrix to prepare CS/STiO_2_ nanocomposite membranes. These sulfonated TiO_2_ particles and CS/STiO_2_ nanocomposite membranes were characterized by Fourier transform infrared (FTIR) spectroscopy, energy-dispersive X-ray (EDX) spectroscopy, X-ray diffraction (XRD) analysis, scanning electron microscopy (SEM), and thermogravimetric analysis (TGA). The effects of sulfonated TiO_2_ particles (STiO_2_) on the physicochemical properties of CS/STiO_2_ membranes, including water uptake, area swelling, ion exchange capacity (IEC), and proton conductivity, were investigated. The interfacial interactions among the –SO_3_H groups of STiO_2_ and the –NH_2_ groups of CS could offer uninterrupted proton conducting pathways, making it possible to enhance proton conductivity along with the mechanical stability of membranes.

## 2. Experimental

### 2.1. Materials and Chemicals

CS with a deacetylation value of 80% and rutile-type titanium dioxide powder with a particle size of 80 nm were purchased from Aladdin Chemical Reagent Co., Shanghai, China. Acetic acid (99%), sulfuric acid (98%) and 1, 3-propane sultone were purchased from Macklin Biochemical Co., Ltd., (Shanghai, China). Absolute ethyl alcohol ≥99.8% and deionized water were used during the experiments.

### 2.2. Sulfonation of Nano-TiO_2_

**Figure 1:**
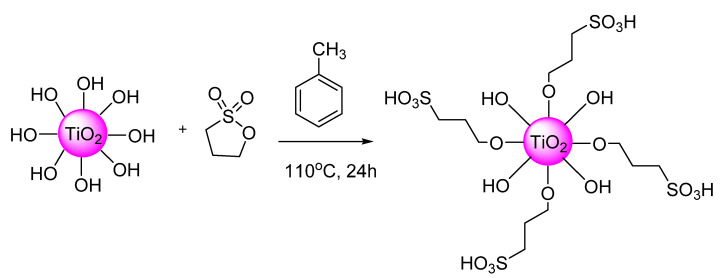
Schematic for the sulfonation of nano-TiO_2_.

One gram TiO_2_ was dispersed in 22 mL of a mixture solution consisting of 1, 3-propane sultone and toluene with a volumetric ratio of 1: 11, and was refluxed at 110 °C for 24 h (Figure 1). The sulfonated titania (STiO_2_) was centrifuged, washed several times with water and absolute ethyl alcohol, and then dried at 80 °C for 24 h.

### 2.3. Preparation of CS/STiO_2_ Nanocomposite Membranes

CS/STiO_2_ nanocomposite membranes with different STiO_2_ contents were fabricated as follows: 1.5 g of CS was dissolved in 2.0 wt% aqueous acetic acid solution under constant stirring, and then a certain amount of STiO_2_ was ultrasonically distributed in 2 wt% acetic acid solution at 300 W and 40 Hz for 1 h. After that, the two mixtures were homogenously mixed at 80 °C for 1 h. After degassing, the homogenous mixture was cooled down without phase separation and sedimentation and poured in a clean glass plate. The contents of the glass plate were dried in an oven at 40 °C for 24 h, and the obtained membranes were cross-linked with 1 M H_2_SO_4_ solution for 48 h to obtain completely cross-linked membranes. Finally, the CS/STiO_2_ membranes were thoroughly washed with deionized water to remove the remaining H_2_SO_4_ and dried under vacuum at 25 °C for 24 h. The CS/STiO_2_ nanocomposite membranes with 1 wt%, 3 wt%, 5 wt% and 7 wt% of STiO_2_ were named as CS/STiO_2_-1, CS/STiO_2_-3, CS/STiO_2_-5 and CS/STiO_2_-7, respectively. For comparison, pure CS membranes were prepared by the same method without the addition of STiO_2_. The thickness of the prepared membranes had range from 20–25 μm.

### 2.4. Characterization

The FTIR spectra of STiO_2_ and CS/STiO_2_ nanocomposite membranes were recorded in transmittance mode at a scan rate of 4 cm^−1^ and a wavelength of 500–4000 cm^−1^ at room temperature using a Nicolet 6700 FTIR spectrometer (Thermo Scientific, Waltham, MA, USA). The phase identification and crystalline structure of CS/STiO_2_ nanocomposite membranes were determined using a Rigaku D/max diffractometer, Rigaku, Tokyo, Japan (2550 VB/PC: CuKα; λ = 0.154 nm radiation) at an operating current and voltage of 40 mA and 40 kV respectively in the 2θ range from 5° to 80° at a scanning speed of 50 min^−1^. The cross-sectional morphologies of CS/STiO_2_ nanocomposite membranes were characterized by SEM (FE-S4800, Hitachi, Tokyo, Japan) equipped with an EDX spectroscope at an accelerating voltage of 200 kV. Prior to analysis, all samples were fractured in liquid nitrogen and sputtered with a thin gold layer. TGA was performed using a TA 50 thermogravimetric analyzer (Shimadzu, Tokyo, Japan) under nitrogen atmosphere at a rate of 10 °C min^−1^ from 25 °C to 700 °C. The mechanical properties were determined using a MTS E43 universal testing machine (MTS, Shanghai, China) according to Chinese standard GB.T.1040.3 at an initial speed of 2 mm/min at room temperature.

### 2.5. Measurement of Water Uptake and Swelling in Dimension

The water uptake of CS/STiO_2_ nanocomposite membranes was measured by the weight difference between dry membranes (W_dry_) and membranes dipped in water for 24 h at room temperature (W_we*t*_). In order to measure swelling in dimension, a piece of dry CS/STiO_2_ nanocomposite with an area of 2.0 cm × 1.0 cm (A_dry_) was immersed in water for 24 h at room temperature and then its area was re-measured (A_wet_, cm^2^), as shown in Equation (2).

(1) $$\mathrm{Water}\;\mathrm{Uptake}\;(\%)=\frac{\;\mathrm{Wwet}\;-\;\mathrm{Wdry}\;}{\mathrm{Wdry}}\text{× }100\;\%\;$$

(2) $$\mathrm{Swelling}\;\mathrm{in}\;\mathrm{dimension}\;(\%)=\frac{\;\mathrm{Awet}\;-\;\mathrm{Adry}\;}{\mathrm{Adry}}\text{× }100\;\%\;$$

### 2.6. Calculation of Ion Exchange Capacity (IEC)

The IECs of CS/STiO_2_ nanocomposite membranes were calculated by classical acid-base titration method. A pre-weighted membrane was dipped in saturated 2.0 M NaCl solution for 48 h to achieve complete exchange of H^+^ with Na^+^. The solution was then titrated by 0.01 M NaOH solution using phenolphthalein as the indicator at room temperature. The IEC value was calculated from Equation (3).

(3) $$\mathrm{IEC}(\mathrm{mmol}/g)=\frac{0.01\;\times \;1000\;\times {\;V}_{\mathrm{NaOH}}\;}{\mathrm{Wdry}}$$

where V_NaOH_ is the volume (mL) of the NaOH solution, and W_dr**y**_ is the weight of the dry membrane.

### 2.7. Measurement of Proton Conductivity

The proton conductivity of CS/STiO_2_ nanocomposite membranes was measured by AC impedance spectroscopy on an electrochemical workstation (PARSTAT 2273 AMETEK, Inc., Berwyn, PA, USA) at a frequency of 1 Hz–106 Hz and an oscillating voltage of 20 mV. Before analysis, all nanocomposite membranes were dipped in 0.2 M H_2_SO_4_ for 24 h. The hydrated membranes (2 cm × 0.5 cm) were fitted in among the two electrodes of the polytetrafluoroethylene (PTFE) mould at room temperature with 100% RH. The proton conductivity was calculated from Equation (4).

σ = L/RA(4)

where L (cm), A (cm^2^) and R (Ω) are the thickness, area and resistance of the membrane, respectively.

## 3. Results and Discussions

### 3.1. Characterization of STiO_2_

**Figure 2:**
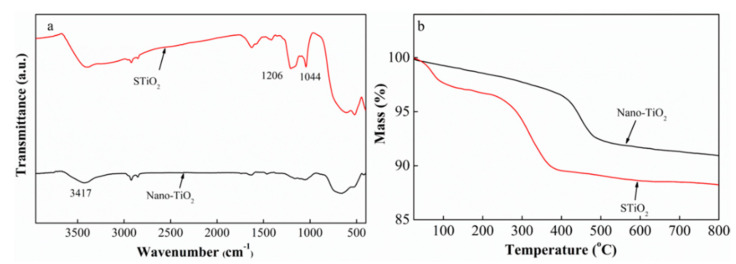
FTIR (**a**) and TGA analysis of TiO_2_ and STiO_2_ particles (**b**).

The surface modification of TiO_2_ was characterized by FTIR, TGA and EDX. The characteristic bands at 1631 and 3417 cm^−1^ in Figure 2a are due to the –OH bending and stretching vibration, respectively. STiO_2_ shows two new bands at 1044 cm^−1^ and 1206 cm^−1^, suggesting the successful grafting of sulfonic acid groups onto TiO_2_ with a significant reduction in hydroxyl groups [44].

The TGA curve displays a three-stage weight loss process (Figure 2b and Table 1). The first stage is attributed to the evaporation of physically adsorbed water, while the second stage is due to the loss of organic matter. However, there is a negligible weight loss at above 400 °C. Upon further heating, the weight loss is related to the decomposition of grafted chains. Moreover, STiO_2_ shows a higher weight loss than TiO_2_, suggesting that STiO_2_ has abundant sulfonic acid groups.

The EDX results (Table 2) show that pristine TiO_2_ has no sulfur, whereas STiO_2_ contains sulfur resulting from the reaction with 1,3-propane sultone, suggesting that sulfonic acid groups have been grafted onto the TiO_2_ surface.

### 3.2. Structural Characterization of CS/STiO_2_ Nanocomposite Membranes

**Figure 3:**
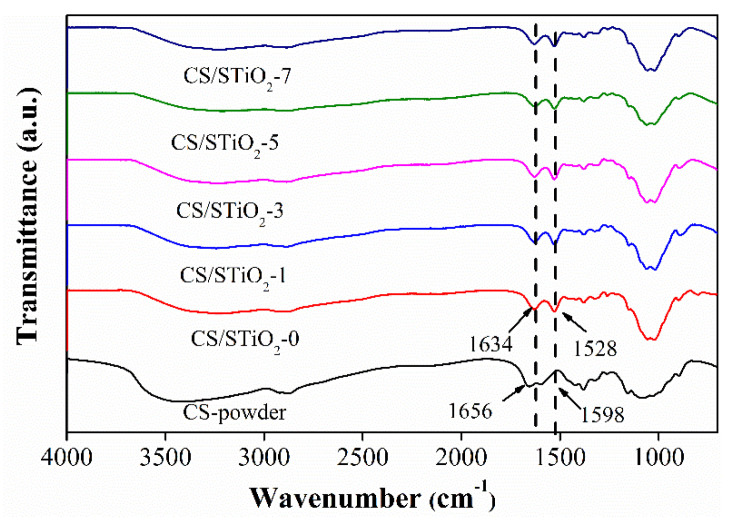
The FTIR spectra of CS control and CS/STiO_2_ nanocomposite membranes.

Figure 3 shows the FTIR spectra of CS/STiO_2_ nanocomposite membranes with different STiO_2_ contents. The characteristic peaks at 1650 cm^−1^ and 1558 cm^−1^ are attributed to the bending vibration of –NH_2_ group. The broad peak at 3426 cm^−1^ is attributed to –OH stretching [45]. The characteristic peaks at 2932 cm^−1^ and 1375 cm^−1^ are attributed to –CH stretching and –CH_3_ symmetric deformation, respectively [46]. After cross-linking with H_2_SO_4_, the characteristic peaks at 1656cm^−1^ and 1598 cm^−1^ are shifted to lower wave numbers, suggesting that the –NH_2_ of CS is protonated. This is endorsed to the shift of hydrogen bond structure and a change in intensity in CS molecules [47]. Enhancing the STiO_2_ content in nanocomposite membranes can shift the bands at 3600–3000 cm^−1^ and 1656–1592 cm^−1^ to lower wave numbers in CS/STiO_2_ nanocomposite membranes. This phenomenon corresponds to the formation of strong interfacial attractions among –SO_3_H of STiO_2_ and –OH/–NH_2_ of CS molecules [48,49,50,51,52].

**Figure 4:**
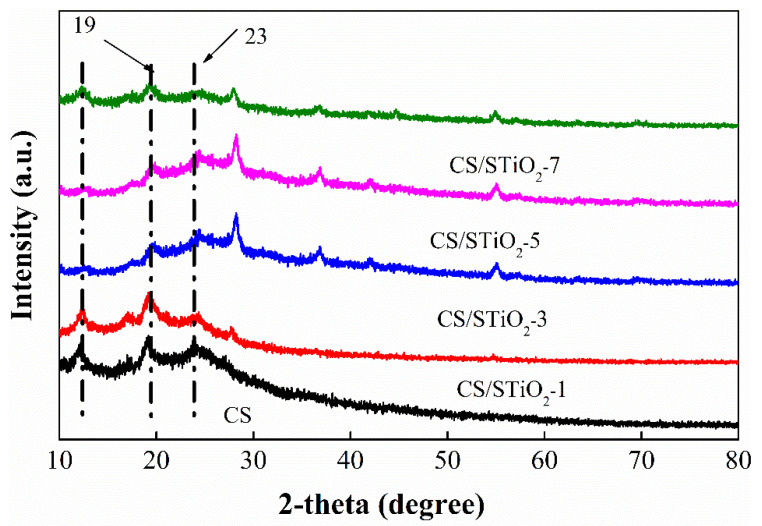
The XRD patterns of CS control and CS/STiO_2_ nanocomposite membranes.

Strong intermolecular and intramolecular interactions within the chitosan molecule is the main reason for its high degree of crystallinity [53,54]. As the proton mobility and diffusion of water molecules in PEMs often take place in an amorphous phase, it is necessary to convert crystalline phase CS into a semi-crystalline or amorphous phase. The impact of STiO_2_ on the crystalline nature of CS was determined by XRD. The characteristic peak at 2θ = 12° depicts the hydrated crystalline section in the CS molecules, whereas that at 2θ = 23° depicts the amorphous section. However, incorporation of STiO_2_ results in a decreased peak intensity at 2θ = 19° and 2θ = 23° (Figure 4). The decrease in the crystalline region is endorsed to the presence of hydrogen and electrostatic interactions between CS molecules and STiO_2_ that can interfere with the order packing of the CS chain and subsequently diminish its crystalline region. The other peaks at 2θ = 27°, 36°, 44° and 56° confirm the presence of rutile phase titania [55].

**Figure 5:**
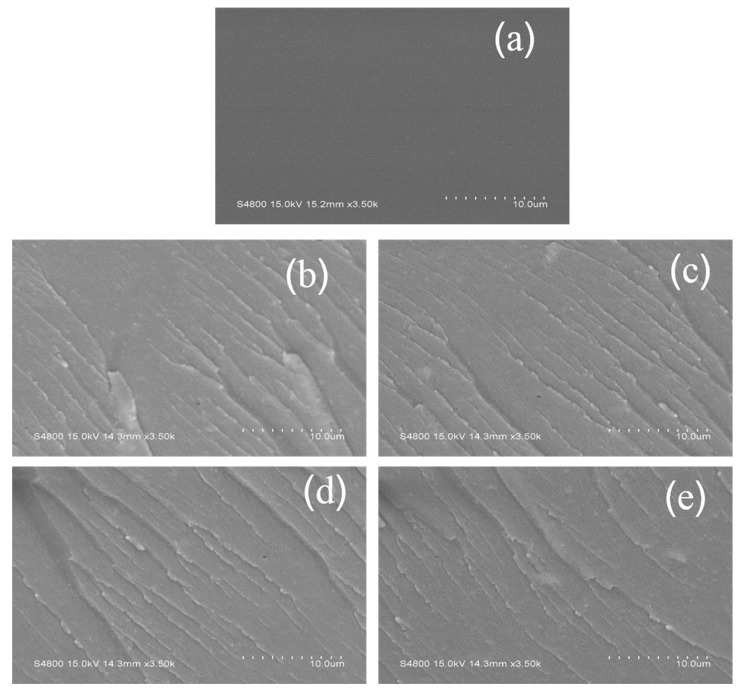
SEM images of cryo-fractured sections of CS control and CS/STiO_2_ nanocomposite membranes with different STiO_2_ contents. CS/STiO_2_–0 (**a**), CS/STiO_2_–1 (**b**), CS/STiO_2_–3 (**c**), CS/STiO_2_–5 (**d**) and CS/STiO_2_–7(**e**).

CS/STiO_2_ nanocomposite membranes were fabricated by solution casting method using CS as the polymer matrix and STiO_2_ as the nanofillers. Figure 5 shows the cross-sectional morphologies of pure CS and CS/STiO_2_ nanocomposite membranes. Pure CS shows a cross-sectional structure with no obvious defects or cracks. However, as the STiO_2_ content increases, a stacked structure is observed in CS/STiO_2_ nanocomposite membranes. Moreover, it is clear that STiO_2_ particles are homogeneously dispersed in the CS matrix with no obvious aggregation, which may be due to the strong electrostatic interactions between the polymer and nanofillers. This results in the formation of uninterrupted proton transfer pathways and consequently an improvement of the proton conductivity of CS/STiO_2_ resultant membranes.

### 3.3. Thermal and Mechanical Stability of CS/STiO_2_ Nanocomposite Membranes

**Figure 6:**
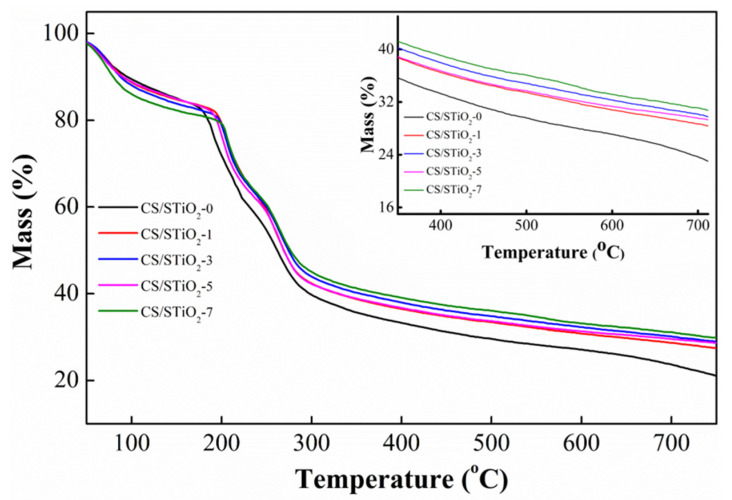
TGA curves of CS control and CS/STiO_2_ nanocomposite membranes.

The thermal stability of CS/STiO_2_ nanocomposite membranes was determined from their TGA thermograms (Figure 6). Pure CS membrane shows a three-stage weight loss process. The initial stage at 30–200 °C is assigned to the evaporation of adsorbed water; the second one at 215–330 °C is due to the degradation of CS side chains; and the last one at 480–800 °C corresponds to the degradation of polymer backbone [49]. However, the incorporation of STiO_2_ can retard the degradation of CS molecules and thus improves the thermal strength of CS/STiO_2_ membranes, which is mainly due to the interfacial interactions (hydrogen bonding or electrostatic interactions) that can interfere with the order packing of CS chains and thus hinder the degradation of polymer backbone of CS molecules. The remaining weight of CS at 750 °C is 21.03%, and as the STiO_2_ content increases from CS/STiO_2_–1 to CS/STiO_2_–7, the residual weight increases from 27.37% to 29.73%.

It is crucial for PEMs to have sufficiently high mechanical stability, which can have dramatic impacts on the durability of PEMFCs. Table 3 shows that all CS/STiO_2_ nanocomposite membranes have higher tensile strength than pristine CS membranes (13.05 MPa). As the STiO_2_ content increases from CS/STiO_2_–1 to CS/STiO_2_–7, the tensile strength increases from 17.84 MPa to 25.30 MPa, which is endorsed to (a) the increase of Coulombic interactions among the amine groups of CS and the –SO_3_H groups of TiO_2_; and (b) the hydrogen bonding between –SO_3_H of STiO_2_ and –OH or –NH_2_ groups of CS. These electrostatic interactions can restrict the mobility of CS chains and subsequently increase the tensile strength of CS/STiO_2_ nanocomposite membranes. The –SO_3_H has a polar and ionic nature, and a proper concentration of sulfonic acid groups can result in the formation of ionic clusters in the CS matrix and consequently an increase in the tensile strength of CS/STiO_2_ nanocomposite membranes. The CS/STiO_2_–7 nanocomposite membrane shows a higher tensile strength than the Nafion 117 membrane (23.6 MPa) [21,46]. The elongation at break (%) of the nanocomposite membranes is also improved compared with that of pure CS membrane. STiO_2_ can halt the propagation of micro-cracks owing to the strong interfacial interactions between STiO_2_ and the CS matrix, thus resulting in an enhancement of the elongation at break of all nanocomposite membranes.

### 3.4. Water and Methanol Uptake, Dimensional Stability and IEC of CS/STiO_2_ Nanocomposite Membranes

**Figure 7:**
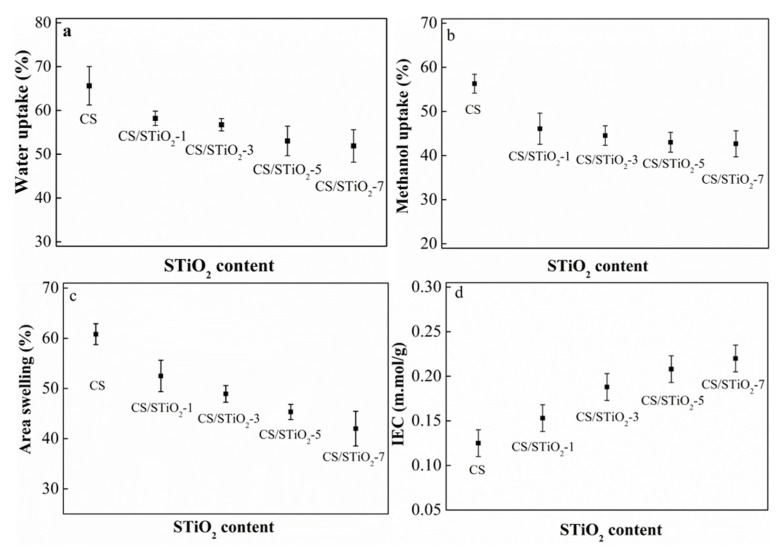
Water uptake (**a**), methanol uptake (**b**), area swelling (**c**) and IEC of CS control and CS/STiO_2_ nanocomposite membranes at room temperature (**d**).

The hydrolytic stability of CS/STiO_2_ nanocomposite membranes was measured to evaluate their applicability in PEMFCs. Water uptake is expected to have significant impacts on the mechanical characteristics and proton conductivity of polymer electrolyte membranes. The existence of water within the membrane can assist the dissociation of various functional groups that is essential to achieve high proton conductivity. However, unnecessary water uptake can lead to dimensional instability that can influence performance of membrane during fuel cell operation. The water uptake of pristine CS membrane is as high as 65% owing to the presence of hydrophilic –OH and –NH_2_ of CS (Figure 7a). However, an increase in STiO_2_ content results in a decrease of water uptake from 58% to 51%. The addition of STiO_2_ rigidifies CS molecules, thus making it less capable of adsorbing solvent molecules [33]. The columbic and hydrogen bonding interactions between the –SO_3_H groups of STiO_2_ and the –NH_2_ and –OH groups of CS can also reduce the water adsorption capacity of CS/STiO_2_ nanocomposite membranes. The incorporation of STiO_2_ can hinder the mobility of CS chains and reduce water storing sites, which results in reduction of the water uptake of CS/STiO_2_ nanocomposite membranes [56,57].

The methanol uptake is also important feature for nanocomposite membranes for their use in PEMFCs. It is desirable for PEMs to have lower methanol absorption, as a high methanol uptake can lead to fuel crossover and thus reduce cell efficiency. Pure CS membrane shows a methanol uptake of 56 %. As the STiO_2_ content increases from CS/STiO_2_-1 to CS/STiO_2_-7, the methanol uptake decreases from 46 % to 42 % (Figure 7b). The methanol uptake of PEMs depends greatly on the available space within membranes. Water is more polar than methanol, making it easier to access the hydrophilic domains in a membrane. As the STiO_2_ content increases, the diffusion capacity of nanocomposite membranes for methanol is suppressed. Hence, the methanol uptake of the nanocomposite membranes decreases [56].

Swelling via hydration can cause dimensional instability in PEMs. The area swelling of CS control and CS/STiO_2_ nanocomposite membranes are shown in Figure 7c. It is noted that the incorporation of STiO_2_ results in decrease in both water uptake and area swelling of the nanocomposite membranes. The swelling of these CS/STiO_2_ nanocomposite membranes can also be reduced, as STiO_2_ can inhibit the swelling of the CS matrix. The CS control membrane shows an area swelling of 60%, whereas that of the CS/STiO_2_–7 membrane is significantly reduced to 42%.

The number of ionizable hydrophilic functional groups in the membrane gives a reliable estimation of proton conductivity. Figure 7d shows that the CS membrane has an IEC value of 0.125 mmol g^−1^. In comparison, increasing the STiO_2_ content from CS/STiO_2_–1 to CS/STiO_2_–7 results in an increase of IEC value from 0.153 to 0.220 mmol g^−1^, which is mainly attributed to the presence of SO_3_H groups in the CS matrix.

### 3.5. Electrochemical Characteristics of CS/STiO_2_ Nanocomposite Membranes

**Figure 8:**
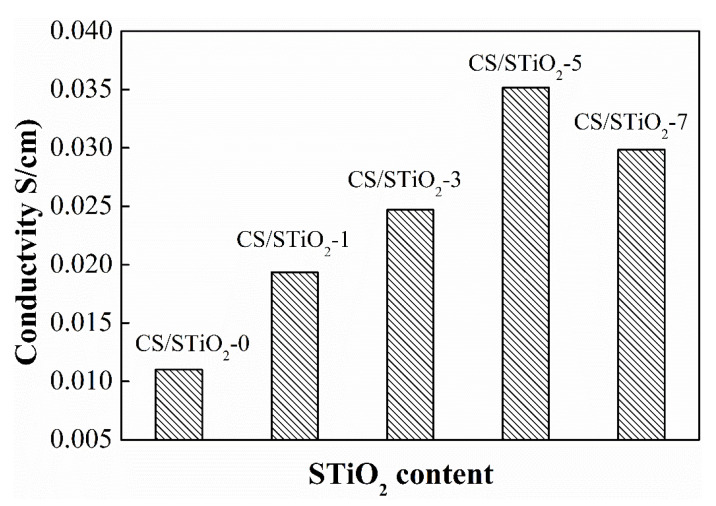
Proton conductivity of CS control and CS/STiO_2_ nanocomposite membranes.

Proton conductivity is mainly determined by water content, distribution of water molecules and the formation of ionic clusters within a PEM [58]. Theoretically, there are two mechanisms for proton transfer in a membrane, including the Vehicle mechanism whereby protons are transported in the form of hydrated hydrogen ions, and the Grotthuss mechanism whereby protons are transported from one carrier to the next through hydrogen bonding networks [59,60]. The CS membrane displays a proton conductivity of 0.011 S·cm^−1^, which is in good agreement with an earlier report [57]. The proton conductivity of Nafion 117 (0.033 S·cm^−1^) is used for comparison in this study [61]. Increasing the STiO_2_ content from 1.0 to 7.0 wt% can increase the proton conductivity from 0.019 to 0.035 S·cm^−1^, as presented in Figure 8. There are abundant sulfonic acid groups in the polymer matrix that can offer extra proton hopping sites. The homogeneous dispersion of STiO_2_ can offer continuous proton conducting pathways through which protons can migrate rapidly. Increasing the STiO_2_ content can also increase the number of ion-exchangeable sites per cluster to improve proton mobility. The generation of acid-base pairs at the CS/STiO_2_ interface can also facilitate the protonation/deprotonation process. All of these could improve the proton conductivity of nanocomposite membranes. The CS/STiO_2_–5 nanocomposite membrane shows a proton conductivity (0.035 S·cm^−1^) equal to that of commercially available Nafion 117 membranes (0.033 S·cm^−1^).

## 4. Conclusions

In this study, a novel one step approach is proposed for the preparation of STiO_2_. CS/STiO_2_ nanocomposite membranes fabricated by the solution casting method for PEMFC applications. These membranes showed lower water and methanol uptake and better dimensional stability than CS membranes. Their thermal and mechanical stabilities can be improved due to the inhibited mobility of CS chains. The CS/STiO_2_–5 membrane showed a higher proton conductivity than the Nafion 117 membrane. These outcomes may probe a simple strategy for the fabrication of chitosan-based PEMs, which has a promising potential in the application of electrochemical devices.

## Figures and Tables

**Table 1:** Thermal properties of TiO_2_ and STiO_2_ from TGA analysis.

Samples	T5% °C	Char Yield (wt. %)			
		500 °C	600 °C	700 °C	800 °C
TiO_2_	420	92	91	91	90
STiO_2_	256	89	88	88	88

**Table 2:** The atomic composition (%) of Ti, O and S in pristine TiO_2_ and STiO_2_.

Serial No.	Ti	O	S
TiO_2_	22.71	77.29	--
STiO_2_	23.43	74.57	2.00

**Table 3:** Mechanical properties of CS control and CS/STiO_2_ nanocomposite membranes.

Membranes	Tensile Strength (MPa)	Elongation at Break (%)
CS/STiO_2_–0	13.05 ± 1.03	15.61 ± 6.98
CS/STiO_2_–1	17.84 ± 2.02	23.54 ± 2.22
CS/STiO_2_–3	21.32 ± 3.33	19.57 ± 4.20
CS/STiO_2_–5	23.27 ± 0.92	21.59 ± 5.70
CS/STiO_2_–7	25.30 ± 2.69	19.34 ± 4.35
Nafion 117	27	–

## Data Availability

The data will be available on the request from corresponding author.

## References

[1] Shen S., Jia T., Jia J., Wang N., Song D., Zhao J., Jin J., Che Q. (2021). Constructing anhydrous proton exchange membranes through alternate depositing graphene oxide and chitosan on sulfonated poly (vinylidenefluoride) or sulfonated poly (vinylidene fluoride-co-hexafluoropropylene) membranes. Eur. Polym. J..

[2] Swaghatha A.A.K., Cindrella L. (2021). Enhanced self-humidification and proton conductivity in magnetically aligned NiO-Co_3_O_4_/chitosan nanocomposite membranes for high-temperature PEMFCS. Polym. J..

[3] Bahar T. (2020). Development of reasonably stable chitosan based proton exchange membranes for a glucose oxidase based enzymatic biofuel cell. Electroanalis.

[4] Hamid A., Khan M., Hussain F., Zada A., Li T., Alei D., Ali A. (2021). Synthesis and physiochemical performances of PVC-sodium polyacrylate and PVC-sodium polyacrylate-graphite composite polymer membrane. Z. Phys. Chem..

[5] Tsen W.-C. (2020). Composite proton exchange membranes based on chitosan and phosphotungstic acid immobilized one-dimensional attapulgite for direct methanol fuel cells. Nanomaterials.

[6] Cui F., Wang W., Shan B., Liu C., Xie C., Zhu L., Chen X., Li N. (2020). Enhanced performance of the chitosan proton exchange membrane via anatase titania anchored go and sodium ligninsulfonate constructing proton transport channels. Energy Fuels.

[7] Wang J., Gong C., Wen S., Liu H., Qin C., Xiong C., Dong L. (2019). A facile approach of fabricating proton exchange membranes by incorporating polydopamine-functionalized carbon nanotubes into chitosan. Int. J. Hydrog. Energy.

[8] Ahmed S., Ali M., Cai Y., Lu Y., Ahmad Z., Khannal S., Xu S. (2019). Novel sulfonated multi-walled carbon nanotubes filled chitosan composite membrane for fuel-cell applications. J. Appl. Polym. Sci..

[9] Ahmed S., Cai Y., Ali M., Khanal S., Xu S. (2019). Preparation and performance of nanoparticle-reinforced chitosan proton-exchange membranes for fuel-cell applications. J. Appl. Polym. Sci..

[10] Sanij F.D., Balakrishnan P., Leung P., Shah A., Su H., Xu Q. (2021). Advanced Pd-based nanomaterials for electro-catalytic oxygen reduction in fuel cells: A review. Int. J. Hydrog. Energy.

[11] Peera S.G., Maiyalagan T., Liu C., Ashmath S., Lee T.G., Jiang Z., Mao S. (2021). A review on carbon and non-precious metal based cathode catalysts in microbial fuel cells. Int. J. Hydrog. Energy.

[12] Lv X.W., Weng C.C., Zhu Y.P., Yuan Z.Y. (2021). Nanoporous metal phosphonate hybrid materials as a novel platform for emerging applications: A critical review. Small.

[13] Shaari N., Kamarudin S.K., Bahru R., Osman S.H., Md Ishak N.A.I. (2021). Progress and challenges: Review for direct liquid fuel cell. Int. J. Energy Res..

[14] Nunes S.P., Ruffmann B., Rikowski E., Vetter S., Richau K. (2002). Inorganic modification of proton conductive polymer membranes for direct methanol fuel cells. J. Membr. Sci..

[15] Yao Z., Zhang Z., Wu L., Xu T. (2014). Novel sulfonated polyimides proton-exchange membranes via a facile polyacylation approach of imide monomers. J. Membr. Sci..

[16] Liao H., Zhang K., Xiao G., Yan D. (2013). High performance sulfonated poly (phthalazinone ether phosphine oxide)s for proton exchange membranes. J. Membr. Sci..

[17] Ahmed S., Cai Y., Ali M., Khannal S., Ahmad Z., Lu Y., Wang S., Xu S. (2019). One-step phosphorylation of graphene oxide for the fabrication of nanocomposite membranes with enhanced proton conductivity for fuel cell applications. J. Mater. Sci. Mater. Electron..

[18] Hamid A., Khan M., Hayat A., Raza J., Zada A., Ullah A., Raziq F., Li T., Hussain F. (2020). Probing the physio-chemical appraisal of green synthesized PbO nanoparticles in PbO-PVC nanocomposite polymer membranes. Spectrochim. Acta Part A Mol. Biomol. Spectrosc..

[19] Jafari Sanjari A., Asghari M. (2016). A review on chitosan utilization in membrane synthesis. ChemBioEng Rev..

[20] Ahmed S., Cai Y., Ali M., Khannal S., Xu S. (2019). Preparation and properties of alkyl benzene sulfonic acid coated boehmite/chitosan nanocomposite membranes with enhanced proton conductivity for proton exchange membrane fuel cells. Mater. Res. Express.

[21] Xiao Y., Xiang Y., Xiu R., Lu S. (2013). Development of cesium phosphotungstate salt and chitosan composite membrane for direct methanol fuel cells. Carbohydr. Polym..

[22] Zada A., Khan M., Hussain Z., Shah M.I.A., Ateeq M., Ullah M., Ali N., Shaheen S., Yasmeen H., Shah S.N.A. (2021). Extended visible light driven photocatalytic hydrogen generation by electron induction from g-C_3_N_4_ nanosheets to ZnO through the proper heterojunction. Z. Phys. Chem..

[23] Huang Z., Guan H.-m., Tan W.l., Qiao X.-Y., Kulprathipanja S. (2006). Pervaporation study of aqueous ethanol solution through zeolite-incorporated multilayer poly (vinyl alcohol) membranes: Effect of zeolites. J. Membr. Sci..

[24] Shaheer Akhtar M., Chun J.-M., Yang O.B. (2007). Advanced composite gel electrolytes prepared with titania nanotube fillers in polyethylene glycol for the solid-state dye-sensitized solar cell. Electrochem. Commun..

[25] Bonderer L.J., Studart A.R., Gauckler L.J. (2008). Bioinspired design and assembly of platelet reinforced polymer films. Science.

[26] Khan R., Dhayal M. (2008). Electrochemical studies of novel chitosan/TiO_2_ bioactive electrode for biosensing application. Electrochem. Commun..

[27] De Sitter K., Winberg P., D’Haen J., Dotremont C., Leiden R., Martens J.A., Mullens S., Maurer F.H.J., Vankelecom I.F.J. (2006). Silica filled poly(1-trimethylsilyl-1-propyne) nanocomposite membranes: Relation between the transport of gases and structural characteristics. J. Membr. Sci..

[28] Yasmeen H., Zada A., Ali S., Khan I., Ali W., Khan W., Khan M., Anwar N., Ali A., Huerta-Flores A.M. (2020). Visible light-excited surface plasmon resonance charge transfer significantly improves the photocatalytic activities of ZnO semiconductor for pollutants degradation. J. Chin. Chem. Soc..

[29] Liu Y., Wang J., Zhang H., Ma C., Liu J., Cao S., Zhang X. (2014). Enhancement of proton conductivity of chitosan membrane enabled by sulfonated graphene oxide under both hydrated and anhydrous conditions. J. Power Sources.

[30] Bai H., Zhang H., He Y., Liu J., Zhang B., Wang J. (2014). Enhanced proton conduction of chitosan membrane enabled by halloysite nanotubes bearing sulfonate polyelectrolyte brushes. J. Membr. Sci..

[31] Wu H., Hou W., Wang J., Xiao L., Jiang Z. (2010). Preparation and properties of hybrid direct methanol fuel cell membranes by embedding organophosphorylated titania submicrospheres into a chitosan polymer matrix. J. Power Sources.

[32] Santamaria M., Pecoraro C.M., Di Quarto F., Bocchetta P. (2015). Chitosan–phosphotungstic acid complex as membranes for low temperature h2–o2 fuel cell. J. Power Sources.

[33] Geng J., Jiang Z., Wang J., Shi Y., Yang D., Xiao L. (2010). Chitosan/titanate nanotube hybrid membrane with low methanol crossover for direct methanol fuel cells. Chem. Eng. Technol..

[34] Wu H., Zheng B., Zheng X., Wang J., Yuan W., Jiang Z. (2007). Surface-modified y zeolite-filled chitosan membrane for direct methanol fuel cell. J. Power Sources.

[35] Wang J., Zhang H., Jiang Z., Yang X., Xiao L. (2009). Tuning the performance of direct methanol fuel cell membranes by embedding multifunctional inorganic submicrospheres into polymer matrix. J. Power Sources.

[36] Wang K., McDermid S., Li J., Kremliakova N., Kozak P., Song C., Tang Y., Zhang J., Zhang J. (2008). Preparation and performance of nano silica/nafion composite membrane for proton exchange membrane fuel cells. J. Power Sources.

[37] Bai H., Li Y., Zhang H., Chen H., Wu W., Wang J., Liu J. (2015). Anhydrous proton exchange membranes comprising of chitosan and phosphorylated graphene oxide for elevated temperature fuel cells. J. Membr. Sci..

[38] Jin Y.G., Qiao S.Z., da Costa J.C.D., Wood B.J., Ladewig B.P., Lu G.Q. (2007). Hydrolytically stable phosphorylated hybrid silica for proton conduction. Adv. Funct. Mater..

[39] Khan M., Hamid A., Tiehu L., Zada A., Attique F., Ahmad N., Ullah A., Hayat A., Mahmood I., Hussain A. (2020). Surface optimization of detonation nanodiamonds for the enhanced mechanical properties of polymer/nanodiamond composites. Diam. Relat. Mater..

[40] Lee W.S., Kang T., Kim S.-H., Jeong J. (2018). An antibody-immobilized silica inverse opal nanostructure for label-free optical biosensors. Sensors.

[41] Chen T.P., Lin C.W., Li S.S., Tsai Y.H., Wen C.Y., Lin W.J., Hsiao F.M., Chiu Y.P., Tsukagoshi K., Osada M. (2018). Self-assembly atomic stacking transport layer of 2d layered titania for perovskite solar cells with extended UV stability. Adv. Energy Mater..

[42] Kharel P.L., Zamborini F.P., Alphenaar B.W. (2018). Enhancing the photovoltaic performance of dye-sensitized solar cells with rare-earth metal oxide nanoparticles. J. Electrochem. Soc..

[43] Khan W.A., Arain M.B., Bibi H., Tuzen M., Shah N., Zada A. (2020). Selective electromembrane extraction and sensitive colorimetric detection of copper(II). Z. Phys. Chem..

[44] Yagizatli Y., Ulas B., Cali A., Sahin A., Ar I. (2020). Improved fuel cell properties of nano-TiO_2_ doped poly (vinylidene fluoride) and phosphonated poly (vinyl alcohol) composite blend membranes for PEM fuel cells. Int. J. Hydrog. Energy.

[45] Li J.-F., Xu Z.-L., Yang H., Yu L.-Y., Liu M. (2009). Effect of TiO_2_ nanoparticles on the surface morphology and performance of microporous pes membrane. Appl. Surf. Sci..

[46] Slade S., Smith J., Campbell S., Ralph T., de Leon C.P., Walsh F. (2010). Characterisation of a re-cast composite nafion^®^ 1100 series of proton exchange membranes incorporating inert inorganic oxide particles. Electrochim. Acta.

[47] Tahrim A., Amin I. (2019). Advancement in phosphoric acid doped polybenzimidazole membrane for high temperature pem fuel cells: A review. J. Appl. Memb. Sci. Technol..

[48] Sharma P.P., Kulshrestha V. (2015). Synthesis of highly stable and high water retentive functionalized biopolymer-graphene oxide modified cation exchange membranes. RSC Adv..

[49] Khan M., Zada A., Hayat A., Ali T., Uddin I., Hayat A., Khan M., Ullah A., Hussain A., Li T. (2021). A concise review on the elastomeric behavior of electroactive polymer materials. Int. J. Energy Res..

[50] Hassan M., Afzal A., Tariq M., Ahmed S. (2021). Synthesis of the hyper-branched polyamides and their effective utilization in adsorption and equilibrium isothermal study for cadmium ion uptake. J. Polym. Res..

[51] Vijayalekshmi V., Khastgir D. (2017). Eco-friendly methanesulfonic acid and sodium salt of dodecylbenzene sulfonic acid doped cross-linked chitosan based green polymer electrolyte membranes for fuel cell applications. J. Membr. Sci..

[52] Liu H., Gong C., Wang J., Liu X., Liu H., Cheng F., Wang G., Zheng G., Qin C., Wen S. (2016). Chitosan/silica coated carbon nanotubes composite proton exchange membranes for fuel cell applications. Carbohydr. Polym..

[53] Ioelovich M. (2014). Crystallinity and hydrophility of chitin and chitosan. J. Chem..

[54] Facchinatto W.M., Dos Santos D.M., Fiamingo A., Bernardes-Filho R., Campana-Filho S.P., de Azevedo E.R., Colnago L.A. (2020). Evaluation of chitosan crystallinity: A high-resolution solid-state nmr spectroscopy approach. Carbohydr. Polym..

[55] Nguyen C.-C., Nguyen D.T., Do T.-O. (2018). A novel route to synthesize C/Pt/TiO_2_ phase tunable anatase–rutile TiO_2_ for efficient sunlight-driven photocatalytic applications. Appl. Catal. B.

[56] Seo J.A., Koh J.H., Roh D.K., Kim J.H. (2009). Preparation and characterization of crosslinked proton conducting membranes based on chitosan and pssa-ma copolymer. Solid State Ion..

[57] Zada A., Ali N., Ateeq M., Huerta-Flores A.M., Hussain Z., Shaheen S., Ullah M., Ali S., Khan I., Ali W. (2020). Surface plasmon resonance excited electron induction greatly extends H_2_ evolution and pollutant degradation activity of g-C_3_N_4_ under visible light irradiation. J. Chin. Chem. Soc..

[58] Kim S.H., Mehmood A., Ahn Y., Kim H.-S., Ha H.Y., Kim D., Han O.H. (2016). Proton conductivity improvement of polymer electrolyte membrane using nano-scale explosion of water in the membrane. J. Electroanal. Chem..

[59] Peighambardoust S., Rowshanzamir S., Amjadi M. (2010). Review of the proton exchange membranes for fuel cell applications. Int. J. Hydrog. Energy.

[60] Vilčiauskas L., Tuckerman M.E., Bester G., Paddison S.J., Kreuer K.-D. (2012). The mechanism of proton conduction in phosphoric acid. Nat. Chem..

[61] Liu H., Wang J., Wen S., Gong C., Cheng F., Wang G., Zheng G., Qin C. (2016). Composite membranes of chitosan and titania-coated carbon nanotubes as promising materials for new proton-exchange membranes. J. Appl. Polym. Sci..

